# Will Endoscopic-Ultrasound-Guided Choledocoduodenostomy with Electrocautery-Enhanced Lumen-Apposing Metal Stent Placement Replace Endoscopic Retrograde Cholangiopancreatography When Treating Distal Malignant Biliary Obstructions?

**DOI:** 10.3390/medicina60020220

**Published:** 2024-01-27

**Authors:** Thomas Guilmoteau, Jérémie Albouys, Abdelkader Taibi, Romain Legros, Marion Schaefer, Jérémie Jacques

**Affiliations:** 1 Hepatogastroenterology, Centre Hospitalier Universitaire Dupuytren, 87000 Limoges, France; tguilmoteau@gmail.com (T.G.); jeremie.albouys@chu-limoges.fr (J.A.); abdelkader.taibi@chu-limoges.fr (A.T.); romain.legros@chu-limoges.fr (R.L.); 2 Hepatogastroenterology, Nancy Regional University Hospital Center, 54500 Nancy, France; m.schaefer@chru-nancy.fr

**Keywords:** MDBO, EUS-CDS, EC-LAMS, ERCP

## Abstract

Malignant distal biliary obstructions are becoming increasingly common, especially in patients with cancers of the pancreatic head, despite progress in medical oncology research. ERCP is the current gold standard for management of such strictures, but the emergence of EC-LAMS has rendered EUS-CDS both safe and efficient. It is a “game changer”; originally intended for ERCP failure, two randomised clinical trials recently proposed EUS-CDS as a first-intent procedure in palliative settings. For resectable diseases, the absence of iatrogenic pancreatitis associated with a lower rate of postsurgical adverse events (compared with ERCP) leads us to believe that EUS-CDS might be used in first-intent as a pre-operative endoscopic biliary drainage.

## 1. Introduction

Endoscopic retrograde cholangiopancreatography (ERCP) is the current first-line biliary drainage procedure for patients with malignant distal biliary obstructions (MDBOs). Biliary decompression is mandatory to increase survival, improve quality of life, and allow for oncological treatment [1,2,3]. However, despite recent improvements, ERCP either fails or is initially impossible in 10–25% of such patients [4,5]. Historically, percutaneous transhepatic biliary drainage (PTDB) was the technique of choice when ERCP failed or was impossible to perform. Over the last two decades, endoscopic ultrasound-guided biliary drainage (EUS-BD) has been preferred to PTBD because the safety profile is better and the technical and clinical success rates are similar [6,7,8]. However, the lack of dedicated tools has rendered the procedure difficult for many years, explaining why the method has been offered only at expert centres. The development of electrocautery-enhanced lumen-apposing metal stents (EC-LAMS) has completely revolutionised therapeutic EUS, enabling the rapid democratisation of EUS-guided choledocoduodenostomy (EUS-CDS) [9]. In this review, we describe the technical aspects of EUS-CDS using LAMS to palliate MDBOs after ERCP failure, useful tips for endoscopists, and outcomes. EUS-CDS may serve as a valuable alternative to ERCP, probably even in resectable diseases.

## 2. EUS-CDS

### 2.1. Materials and the Procedure (Video S1)

The first cases of EUS-CDS were described in 2001 by Giovannini using plastic stents. The development of fully and partially covered SEMS permitted the improvement in clinical and technical success of this procedure, with a lower rate of adverse events. Recently, the emergence of LAMS allowed Itoi to perform the first EUS-CDS employing LAMS in 2014 [10]. The most popular EC-LAMS system is the HOT AXIOS stent (Boston Scientific Corporation, Natick, MA, USA); this two-flanged stent is fabricated from nitinol wire and is fully covered; an enhanced electrocautery system allows a “one-step procedure”. The most common contraindications for EUS-CDS with LAMS are a large vessel (commonly associated with portal hypertension) between the common bile duct (CBD) and the duodenum, ascites, refractory coagulopathy, and thrombopenia. Concerning CBD diameter, a diameter of less than 15 mm has been shown to be associated with a higher rate of technical failure and must be considered as a relative contraindication outside of expert hands, and it requires the use of a guidewire.

The procedure must be performed in an operating room equipped with fluoroscopy; indeed, complicated cases and rescue procedures require the use of contrast agent and X-rays. Patients are usually under general anaesthesia or conscious sedation during the procedure. A linear echoendoscope is positioned in the duodenal bulb via a long route; this identifies the CBD. Then, colour doppler imaging is used to locate the vessels and define the best “shooting window”. Usually, this is a dilated region of the CBD (>15 mm in diameter) close to the duodenal wall (within 10 mm). After positioning has been established, the catheter is pushed in the direction of the CBD using cauterisation (a pure-cut current) to create a fistula between the CBD and the duodenum. Then, the fistula is covered with the LAMS and the first (distal) flange is deployed within the biliary tract under ultrasonographic guidance. Then, the second (proximal) flange is released, either by gently pulling the catheter until release is apparent or using the “intra-channel stent release” technique. The presence of bile (that has passed through the LAMS) in the duodenal bulb visually confirms correct stent positioning.

Three different techniques can be used to perform EUS-CDS with EC-LAMS:

1. Puncture of the CBD using a 19-gauge needle and insertion of a 0.025- or 0.035-inch stiff guidewire before EC-LAMS insertion, followed by stent deployment along the guidewire.
2. Direct puncture of a dilated duct with the EC-LAMS device equipped with a pre-loaded guidewire that can be advanced in the biliary tree in case of difficult situations, to secure the field before deploying the stent.
3. Direct puncture of a dilated duct using the EC-LAMS system, followed by direct deployment of the stent without use of a guidewire (the free-hand technique).

**Figure 1:**
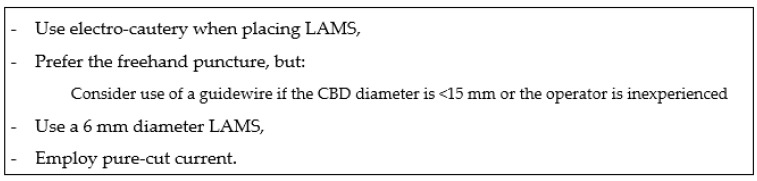
Recommendations for EUS-CDS using LAMS.

We recommend the free-hand technique when the CBD diameter exceeds 15 mm; a 6 or 8 mm LAMS is optimal (Figure 1). A recent case series found that, in univariate analysis, use of a guidewire after fine-needle aspiration puncture of the dilated CBD was a risk factor for technical failure [11,12].

Sometimes, in palliative settings, transduodenal puncture is impossible, whereas transgastric approach seems feasible. In this situation, it is important to ensure that the distance between the gastric wall and common bile duct is less than 10 mm to avoid the risk of secondary LAMS migration.

Binda et al. also described a rescue strategy using EUS-gallbladder drainage (EUS-GBD) when EUS-CDS is not feasible. Either a transgastric or transduodenal approach is possible, and a technical success rate of 100% has been reported on a 48-patient cohort, with a high clinical success rate (81.3%). However, this strategy requires the cystic duct to be permeable at the time of endoscopy [13].

However, when the CBD diameter is 12 to 15 mm, the risk of technical failure increases. In such a situation, the stent should be preloaded using a guidewire to secure the field; this facilitates salvage endoscopic therapy if stent misdeployment occurs. EUS-CDS using LAMS seems “easier”; however, a meta-analysis suggests a comparable efficacy in terms of technical and clinical success whether using an LAMS or an SEMS. EUS-CDS using SEMS therefore could be a valuable alternative especially when CBD is inferior to 15 mm [14].

### 2.2. Expected Early Adverse Events

A recent review of LAMS misdeployment revealed an overall misdeployment rate of 5.8% after EUS-CDS, mostly of the distal flange [15]. Such misdeployments are usually associated with small-diameter CBDs (<15 mm). In such cases, the use of a guidewire allows the fistula to be secured if misdeployment occurs and to place a new self-expanding metallic stent (SEMS) or an LAMS after removal of the first one. If misdeployment is associated with a large CBD, a rapid new CDS procedure using an EC-LAMS is required; bile leakage must be prevented. Such leakage after EUS-CDS using LAMS is usually attributable to proximal flange misdeployment. This is the worst-case scenario; it is essential to place a guidewire within the CBD (through the stent) and then deliver a classic, covered metal stent. Such events can be prevented using the “intra-channel stent release” procedure. When endoscopic management fails (the misdeployment rate is around 10%), a radiological (using PTBD) or surgical intervention is usually needed, unless ERCP is ultimately successful.

Proximal flange misdeployments or migrations can also be managed using endoscopic ultrasound. Sato et al. recently described the “Lambda stenting technique” by puncturing the proximal extremity of the migrated LAMS under endoscopic ultrasound control, allowing the insertion of a guidewire and then the deployment of a new covered SEMS through the initial LAMS [16].

Bleeding is usually preventable when colour Doppler is used to identify interposing blood vessels. Stent self-expansion is haemostatic in most cases. Supportive treatment adequately handles minimal bleeding; major bleeding creates a need for computed tomography possibly followed by radiological arterial embolisation.

Cholangitis after EUS-CDS with LAMS placement usually manifests as fever and cholestasis caused by either stent obstruction or the contrast agent employed during cholangiography. Antibiotics are usually adequate, but if an obstruction develops early (attributable to compression of the biliary side or food impaction), endoscopic reintervention may be necessary, possibly using co-axial double-pigtail plastic stents (DPPSs) [17,18] (Table 1).

## 3. Outcomes of LAMS

### 3.1. LAMS after ERCP Failure

EC-LAMS simplifies and generalises EUS-CDS use after ERCP failure, principally caused by duodenal obstructions and cannulation issues. Many studies have retrospectively evaluated the outcomes afforded by LAMS in such situations; EUS-guided choledocoduodenostomy affords an excellent technical success rate (88.5–100%) and a low early adverse event rate (0–15.8%) usually without any need for additional surgery. The clinical success rate is high (79–100%); it directly depends on the technical success and allows a quick oncologic management of patients. The LAMS dysfunction rates during follow-up range from 6.7% to 31.8% (Table 2) [11,12,18,19,20,21,22,23].

Of the 734 procedures performed in the eight studies cited above, no post-procedural pancreatitis was observed. Bile leakage was very rare and occurred only after LAMS misdeployment or early stent migration, in marked contrast to the high rates of previous techniques using plastic stents or non-dedicated SEMSs [24,25,26]. In terms of recurrent biliary obstruction (RBO) after EUS-CDS using EC-LAMS, one meta-analysis of five studies (201 patients) reported an RBO rate of 11.3% [27], mostly caused by food particles or stones. Sump syndrome, LAMS migration, tumoral obstruction, and impaction of the distal flange on the opposite biliary wall have also been described. However, the only first-intent prospective study (from the Netherlands) reported a 55% stent dysfunction rate during follow-up [28], much higher than reported in other studies. The cited authors suggested that stent dysfunction was under-evaluated in retrospective studies. However, the classic 10–15% dysfunction rate was confirmed in the LAMS groups of two recent randomised controlled trials (RCTs) that compared EUS-CDS with EC-LAMS and ERCP [29,30]. Vanella et al. reported a rate of stent dysfunction of 31.8%, mostly from stone or food impaction (up to 52% of dysfunction causes). LAMS obstructions were observed in 14.8%, mostly on the duodenal side (attributable to tumour progression). Gastric outlet obstructions (GOOs) were noted in 26% of cases, 96% of whom were treated endoscopically; only one case required PTBD. Food or stone impactions have commonly been managed using balloon or basket devices, followed by coaxial double-pigtail stent (DPPS) placement. If LAMS obstructions are caused by tumour progression, placement of a DPPS or SEMS across the LAMS is a good option; if this fails, a more advanced endoscopic procedure such as EUS-hepaticogastrostomy (HGS) or transpapillary SEMS placement after establishment of a “through-LAMS rendezvous” may be required [19]. In a recent single-centre prospective study on 123 patients, we found that the presence of a duodenal stent and a main bile duct diameter < 15 mm were significant risk factors for RBO during follow-up [31]. To reduce the RBO rate and the need for recurrent biliary intervention (RBI), some authors suggest that DPPSs should routinely be inserted through the LAMS [17]. This might reduce the rate of LAMS migration, food and stone impactions, tumoral obstructions, and sump syndrome by maintaining the LAMS axis vertical within the bile duct, and it might also reduce impaction of the distal flange against the opposite biliary wall. El Chafic et al. reported a significantly lower RBO rate (12% vs. 50%) in patients for whom DPPSs were inserted via the LAMS than not. On et al. reported lower rates (6.3% vs. 12.2%) of cholangitis and RBO (0% vs. 12.2%) in patients with DPPSs [18,20]. The large, multicentre, prospective Biliary-Apposing Metal Pigtail (BAMPI) RCT is currently assessing the outcomes of routine addition of a coaxial axis-orienting DPPS through the LAMS; this seeks to prevent RBO [32]. As EUS-CDS is a supratumoral drainage route, this should reduce the rate of tumoral obstruction, but to date, there is no evidence indicating that EUS-CDS stent patency is longer than that of a transpapillary SEMS.

The safety of EUS biliary drainage procedures has recently been studied in a large meta-analysis of 155 studies including 7887 patients. EUS-CDS using LAMS is associated with a global adverse event rate of 9.7%, less than 1% of severe adverse events, no procedural mortality, and almost no risk of procedure-related pancreatitis (0% [0–0.4%]) [33].

### 3.2. LAMS Versus ERCP: Two RCTs

Two recent RCTs compared ERCP and EUS-CDS (using LAMS) for management of unresectable MDBOs [29,30]. Both works compared the 1-year permeabilities associated with the two techniques; these were 87.9–90.1% for ERCP vs. 88.2–90.4% for EUS-CDS, essentially the same. However, the technical success rate of EUS-CDS (90.4–96.2%) was significantly better than that of ERCP (76.3–83.1%); the clinical success rates were similar. Importantly, in a Canadian study, EUS-CDS was performed by endoscopists with limited experience (two procedures or less), whereas ERCP was performed by highly trained endoscopists (250 to 1000 procedures), confirming the relative simplicity of EUS-CDS compared to ERCP. Despite the limited experience of EUS-CDS endoscopists, EUS-CDS durations were significantly shorter than those of ERCP in both studies (10 to 14 min for EUS-CDS vs. 23 to 25 min for ERCP); the adverse event rates were similar (Table 3). No pancreatitis was observed in the EUS-CDS arms. In addition, we feel it is important to point out that Chen et al. reported, in the ERCP arm, a high rate of recourse to advanced biliary access techniques, such as pre-cutting (40%).

These two studies suggest that EUS-CDS is technically easier than ERCP, requires less time, affords similar clinical success and 1-year patency rates, and has a similar safety profile. Cost-effectiveness data are not yet available but LAMS costs will soon decrease due to industrial competition and the increased indications for the procedure. The high cost may currently limit accessibility, especially if resources are constrained. Future cost-effectiveness analyses can use the data generated by these two randomised studies including details such as procedure time, adverse events, and their management, as well as long-term follow-up results, which must be adapted to the specific health care system in order to be used by decision makers.

Although even nonexpert endoscopists can readily perform EUS-CDS using EC-LAMS, we strongly recommend that all operators be trained in ERCP and guidewire exchange. The (rare) LAMS misdeployments require the use of a guidewire-directed biliary device or a switch to classical ERCP or duodenal closure of the perforation. Neither study cited above indicated that the primary outcomes of EUS-CDS were better than those of ERCP. However, the high technical and clinical success rates obtained by “inexperienced” Canadian endoscopists, the halving of procedural duration at a time when endoscopy units are running at full capacity, and the overall better safety profile will encourage centres to switch to initial EUS-CDS with LAMS when treating distal biliary obstructions.

These outstanding results from these two randomised trials confirm the recent recommendations of the European Society of Digestive Endoscopy [5], positioning EUS-CDS with EC-LAMS as a potential first-line alternative to ERCP in cases of distal malignant biliary obstruction, particularly in instances of the main bile duct dilation exceeding 15 mm and pre-procedure risk factors for failure.

### 3.3. EUS-CDS Using LAMS and Duodenal Obstructions

A retrospective, single-centre study of 63 patients with unresectable pancreatic head cancers and on either chemotherapy or radiotherapy showed that 38% developed symptomatic duodenal obstructions [34]; the rate is increasing despite recent developments in oncological therapies. Three distinct obstruction onsets have been described: GOO before MDBO, after MDBO, or concomitant with MDBO. The most frequent cause of both duodenal and biliary obstructions is pancreatic head cancer. Three levels of GOO are known: type 1 (GOO on the bulb or upper duodenal genu; the papilla is not involved), type 2 (GOO in the second part of the duodenum; the papilla is involved), and type 3 (GOO in the distal part of the duodenum) [35]. GOOs (usually types 1 and 2) cause >25% of all recurrent jaundice cases after EUS-CDS, attributable to food impaction, reflux cholangitis, or tumoral invasion of the LAMS. Thus, GOO resolution is essential for successful biliary drainage. When placing enteric stents, EUS-CDS is superior to transpapillary SEMS placement in terms of both technical success and clinical success, and stent patency tends to be better [36,37]. Debourdeau et al. recently showed that the contemporaneous treatment of duodenal and biliary obstructions is not associated with a higher rate of adverse events than having two endoscopic procedures for these issues, and it reduced the hospital stay (most patients underwent EUS-HGS, CPRE, or PTBD associated with enteral stenting) [38].

However, a duodenal obstruction with or without an enteral stent is a risk factor for biliary LAMS obstruction as confirmed by the retrospective, international CABRIOLET study [39]. The technique used for duodenal obstruction treatment (enteral stent or EUS-gastroenterostomy (EUS-GE)) is at least as important as the technique used for biliary obstruction treatment (transpapillary SEMS, EUS-CDS, or EUS-HGS) in order to avoid recurrent biliary obstructions. A recent randomised clinical trial suggests that EUS-GE, in expert hands, reduces the frequency of reintervention and improves stent patency, with better patient-reported outcomes, in comparison with enteral stent [40].

Double duodenal and biliary obstructions are optimally treated by combining EUS-GE with HGS (a double EUS bypass); the rate of later biliary events is low. However, this is technically challenging and patients should be referred to expert centres. Performing an EUS-CDS with LAMS with the addition of DPPS could be a more available and easier solution. However, suboptimal biliary drainage is unacceptable; recurrent biliary obstructions compromise chemotherapy. Additional prospective data are needed to determine the optimal drainage combination for the patient, with the aim of enhancing their nutritional status and achieving the most effective biliary drainage (reducing RBO rate), thereby facilitating the most effective oncological treatment possible.

### 3.4. Use of LAMS for Preoperative Drainage

A few studies have employed EUS-CDS for preoperative drainage. However, many surgeons are concerned that EUS-CDS with LAMS may compromise the outcomes of later pancreaticoduodenectomy. One study on five patients reported excellent results, no severe adverse events, and no impact on the success of later pancreaticoduodenectomy [41]. Such results are supported by a small French series (21 patients) described in 2021; 7 benefited from first-intent EC-LAMS and 14 from EC-LAMS after ERCP failure (Table 4) [42]. Importantly, LAMS was not associated with higher rates of pre- or postsurgical adverse events. Recently, we retrospectively compared surgical outcomes after biliary drainage using EUS-CDS, SEMS placement, or ERCP. Post-EUS-CDS pancreatitis was absent, and the postsurgical adverse event rate was lowest after EUS-CDS with LAMS placement. Jaundice resolution was also more rapid; preoperative chemotherapy could thus commence earlier [43].

These data are, of course, preliminary and must be confirmed through prospective and randomised studies. However, it is important for the community to be aware that the use of EUS-CDS with LAMS does not appear to impact surgical procedures in cases of resectable disease. The absence of acute pancreatitis risk, which could contraindicate surgical management with this procedure, is an undeniable advantage over ERCP. Consequently, it is legitimate to question the role of EUS-CDS with EC-LAMS as a first-line approach in this common clinical situation.

A prospective, multicentre study in France will soon commence and will compare the preoperative biliary-drainage-related complications between SEMSs and LAMSs.

## 4. Conclusions: Will EUS-CDS with LAMS Replace ERCP for MDBO Patients?

EUS-CDS has revolutionised the management of malignant bile duct obstructions, especially when ERCP has failed. The new electrocautery-enhancing delivery system has greatly simplified the procedure, especially for nonexpert endoscopists; the learning curve plateaus earlier than that of ERCP. Severe adverse events are rare, but the management of stent misdeployment requires advanced endoscopic skills (particularly ERCP). Stent patency does not seem to be any longer than that of SEMS, and it remains unclear whether the routine addition of DPPS improves patency in patients with recurrent jaundice or prevents RBO.

In palliative settings, recent European guidelines have confirmed that EUS-CDS with LAMS should be performed first at expert centres, particularly in cases for whom biliary drainage is difficult, provided that the bile duct diameter is >15 mm. In our opinion, ERCP should be preferred when the diameter is <15 mm because of the lower risks for adverse events and RBOs. Duodenal obstructions cause most failures of ERCP procedures that seek to treat MDBOs. EUS-CDS is inappropriate for such patients; they require (difficult) double EUS bypasses. In terms of preoperative biliary drainage, more data are urgently needed; however, the available data suggest that EUS-CDS with LAMS placement does not compromise surgical outcomes if ERCP fails.

Biliary drainage needs to be effective over time and comprehensive large-scale prospective data are required to accurately identify risk factors for obstructions that might favour an alternative biliary drainage approach.

Ultimately, any decision should be based on a case-by-case assessment of patient factors, the endoscopist’s expertise, and resources. EUS-CDS may not entirely replace ERCP but may play an ever more significant role in the management of distal MBOs. The management of biliary obstruction is an integral part of oncological treatment in cases of obstructive pancreatic cancer. The biliopancreatic endoscopist must master both EUS and ERCP biliary drainages in order to choose the best drainage route regarding patient situations and to propose the most satisfactory oncological treatment.

## Figures and Tables

**Table 1:** Early adverse events and their management.

Type of AE	Prevention	Management
Cholangitis	Avoid contrast agent use	Antibiotics Endoscopic reintervention (DPPS or SEMS?)
Distal flange misdeployment	CBD diameter > 15 mm	*Without guidewire:* removal of LAMS + duodenal closure (OTSC) +/− repeat procedure or transpapillary SEMS after ERCP PTBD
	If <15 mm, use of a guidewire procedure	*With a guidewire:* removal of LAMS +/− cSEMS +/− repeat LAMS PTBD
Proximal flange misdeployment	«Intra-channel release» technique	Bridging-covered stent (under direct visualisation or EUS-guided) or PTBD
Bleeding	Color doppler Monitoring of coagulation function	Supportive management In case of major bleeding, consider arterial embolisation

**Table 2:** Retrospective studies on EUS-CDS performed after ERCP failure to palliate MDBOs.

Author [Ref]	Patients	TS	CS	Bile Duct Diameter	Freehand	Mean Follow-Up	Stent Obstruction	Early AE	Type of Stent Used
Year									
**Kunda et al.** [21]	57	98.2%	96.4%	17.9 mm	None	151 d	9.3%	7%	Axios and Hot Axios
2016									6 × 8 mm (64.2%) 8 × 8 mm (3.6%)
									10 × 10 mm (28.6%) 15 × 10 mm (3.6%)
**Jacques et al**. [12]	52	88.5%	100%	17.2 mm	94.2%	157 d	11.5%	3.8%	Hot Axios
2019									6 × 8 mm (82.7%) 8 × 8 mm (13.5%) 15 × 10 mm (3.8%)
**Tsuchiya et al.** [22] *	19	100%	79%	17.3 mm	5.2%	145 d	26.3%	15.8%	Hot Axios
2018									6 × 8 mm (52.7%) 8 × 8 mm (47.3%)
**El Chafic et al.** [18]	67	95.5%	100%	17.6 mm	None	119 d	17.5%	7.5%	Hot Axios
2019									10 × 10 mm (100%)
**Jacques et al.** [11]	70	98.6%	98.6%	17.7 mm	90%	153 d	10%	0%	Hot Axios
2020									6 × 8 mm (85.7%) 8 × 8 mm (13%) 10 × 10 mm (1.3%)
**Fugazza et al.** [23]	256	93.3%	96.2%	17.3 mm	94.5%	151 d	6.7%	2.7%	Hot Axios
2022									6 × 8 mm (33.6%) 8 × 8 mm (51.6%)
									10 × 10 mm (10.9%) 15 × 10 mm (2.7%)
									Nagi Stent (1.2%)
**On et al.** [20]	120	90.8%	94.8%	18.7 mm	71.4%	70 d	8.3%	8.3%	Hot Axios
2022									6 × 8 mm (38.7%) 8 × 8 mm (57.1%) 10 × 10 mm (4.2%)
**Vanella et al.** [19]	93	97.9%	93.4%	-	98.9%	138 d	31.8%	9.7%	Hot Axios
2023									6 × 8 mm (66.7%) 8 × 8 mm (32.3%) 15 × 10 mm (1%)

* prospective study.

**Table 3:** RCTs comparing EUS-CDS and ERCP for first-line palliation of MDBOs.

Author [Ref]		Patients	TS	CS	1-Year Patency	Time to RBO	Bile Duct Diameter	Procedure Duration	Early AE	Minimal Experience
Year										
**Chen et al.** [30]	**ERCP**	71/144	83.1%	85.9%	90.1%	200.1 d	18 mm	23.1 min	12.7%	1000
2023	**EUS-CDS**	73/144	90.4%	84.9%	90.4%	163.9 d	17.7 mm	14 min	12.3%	2
**Teoh et al.** [29]	**ERCP**	76/155	76.3%	90.8%	87.9%	183.2 d	16.8 mm	25 min	17.1%	250
2023	**EUS-CDS**	79/155	96.2%	93.7%	88.2%	161.3 d	15.9 mm	10 min	16.5%	20

**Table 4:** Outcomes when LAMS is used for preoperative drainage.

Author [Ref]	Study Type	Patients	TS	CS	Bile Duct Diameter	Procedure Duration	Direct Puncture	Early AE	Type of Stent Used
**Year**									
**Fabbri et al.** [41]	Retrospective	5	100%	100%	20.2 mm	6 min	100%	0%	Hot Axios
**2019**									(8 × 8 mm (80%), 10 × 10 mm (20%))
**Gaujoux et al.** [42]	Retrospective	21	100%	100%	-	-	-	0%	Hot Axios
**2021**									(6 × 8 mm (95%) or 8 × 8 mm (5%))
**Janet et al.** [43]	Retrospective	44	100%	89.3%	-	-	100%	17.9%	Hot Axios
**2023**									(6 × 8 mm or 8 × 8 mm)

## Supplementary Materials

The following supporting information can be downloaded at https://www.mdpi.com/article/10.3390/medicina60020220/s1: Video S1: EUS-choledocoduodenostomy.
